# Commercial Methods for Antifungal Susceptibility Testing of Yeasts: Strengths and Limitations as Predictors of Resistance

**DOI:** 10.3390/jof8030309

**Published:** 2022-03-17

**Authors:** Ana Espinel-Ingroff

**Affiliations:** VCU Medical Center, Richmond, VA 23219, USA; victoria.ingroff@vcuhealth.org

**Keywords:** antifungal resistance, *Candida* and triazole mutants, echinocandin mutants, ECVs for commercial methods, Etest and SYO ECVs for *Candida* spp.

## Abstract

Susceptibility testing can yield variable results because it is method (commercial or reference), agent, and species dependent. Therefore, in order for results to be clinically relevant, MICs (minimal inhibitory concentrations) or MECs (minimal effective concentrations) should help in selecting the best treatment agent in the clinical setting. This is accomplished by categorical endpoints, ideally, breakpoints (BPs) and/or ECVs/ECOFFs (epidemiological cutoff values). BPs and ECVs are available by the reference methods (CLSI [Clinical and Laboratory Standards Institute] and EUCAST [European Committee on Antifungal Susceptibility Testing]) for a variety of species/agent combinations. The lack of clinical data precludes establishment of BPs for susceptibility testing by the commercial methods and ECVs have only been calculated for the Etest and SYO assays. The goal of this review is to summarize the variety of commercial methods for antifungal susceptibility testing and the potential value of Etest and SYO ECVs for detecting mutants/non-wild type (NWT) *Candida* isolates. Therefore, the literature search focused on publications where the commercial method, meaning MICs and ECVs, were reported for specific NWT isolates; genetic mutations have also been listed. For the Etest, the best performers recognizing the NWT were anidulafungin ECVs: 92% for the common species; 97% for *C. glabrata* and fluconazole ECVs, mostly for *C. parapsilosis* (45 NWT isolates). By the SYO, posaconazole ECVs recognized 93% of the *C. albicans* and 96% of the *C. parapsilosis* NWT isolates and micafungin ECVs 94% (mostly *C. albicans* and *C. glabrata*). Smaller sets, some with clinical data, were also listed. These are promising results for the use of both commercial methods to identify antifungal resistance (NWT isolates). However, ECVs for other species and methods need to be defined, including the *C. neoformans* complex and emerging species.

## 1. Introduction

Standardized broth microdilution methods and breakpoints (BPs) have been developed for in vitro antifungal susceptibility testing by both the Clinical and Laboratory Standards Institute [CLSI] and The European Committee on Antifungal Susceptibility Testing [EUCAST]) [1,2]. In addition, ECVs/ECOFFs (epidemiological cutoff values) have also been established [2,3,4]. In resource settings with limited access to reference testing systems, the accurate species-level identification of bloodstream *Candida* and other species isolates and the susceptibility testing of first-line agents (the polyenes, triazoles, and echinocandins) is often problematic. However, across the years, several commercial susceptibility methods have been used in clinical and research laboratories. These methods provide minimal inhibitory concentrations/minimal effective concentrations (MICs/MECs) and could be helpful in such limited settings. In order to be useful in clinical laboratories, commercial antifungal susceptibility methods have been compared with the reference assays in two main ways [5,6]. The first type of comparison (essential agreement [EA]) provided the index of “reproducibility” between reference and the commercial method results or the percentage of agreement between MICs/MECs by both methods. However, the most critical evaluation, designated as categorical agreement (CA), compared commercial and reference MIC/MEC data as the percentage of isolates having the same categorization [5,6]. For the Etest, both the EA and CA (defined as a discrepancy in MICs of no more than two doubling dilutions) were recently reviewed [5,6]. The conclusions were ambiguous. The main issue is that the lack of clinical data precludes the definition of BPs for commercial methods, but Etest and SYO ECVs (based solely on in vitro data) have been defined for various combinations of *Candida* and *Aspergillus* spp. versus the triazoles and echinocandins (Table 1) [7,8,9,10]. Below, we provide a brief summary of the sequence of studies that led to the development of current CLSI/EUCAST BPs, while the CA between reference and various commercial methods were being evaluated. The same applies when testing the *C. neoformans* species complex by the commercial methods; both Etest and SYO have been assessed for this purpose since 2008 [11,12,13]. However, ECVs have not been defined for any commercial method for the latter species and the mechanisms of antifungal resistance have not been clearly elucidated for these species. 

## 2. Development of Reference BPs and Early CA Evaluation of Commercial Methods

Between 1997 and 2011, CLSI fluconazole BPs were based on either macro or microdilution MICs [14], and although voriconazole BPs were defined with data by the microdilution method, the results were after 48 h of incubation [15]. The current CLSI species-specific BPs for fluconazole, voriconazole, and the three echinocandins and some *Candida* spp. were established between 2010 and 2011, as listed in the M60 document [16,17,18]. Furthermore, wild-type (WT) MIC distributions, ECVs, and resistance mechanisms became important in BP definition in addition to the correlation of in vitro versus in vivo results from clinical trials. The previous BPs lacked sensitivity in discriminating WT strains of *Candida* from NWT strains (possess mutational resistance mechanisms). Therefore, the CA evaluations of commercial methods prior to 2011 were conducted with either the CLSI macrodilution method or 48 h MICs; however, the current EUCAST fluconazole and voriconazole BPs were established in 2008 [2]. Table 2 provides a list of the commercial methods evaluated between 2002 and 2011 by the “acceptable essential agreement” (EA, MICs within two double fold dilutions) [19,20,21,22,23,24,25,26]. Among those, it is worthwhile to mention two more recent evaluations that included the Vitek method [27,28]. This commercial assay covers both the isolates species identification as well as the susceptibility testing result. In one of those two studies, one echinocandin *FKS* WT and eleven *C. glabrata* mutants, respectively, were evaluated with the Vitek 2 AST-YS06 yeast card [27]. Again, the problem is that ECVs have not been defined for this test against any fungal species.

## 3. Review Purpose and Guidelines

The purpose of this review was to summarize the ability of both Etest and SYO commercial method ECVs in categorizing triazole or echinocandin NWT isolates of the five common *Candida* spp. Since the role of the ECV is to identify mutants or NWT isolates [3,4,29], publications were examined where there was a coupling of resistance mechanisms, MICs by the commercial method evaluated, and ECVs (Etest and SYO) available for the particular species and agent. This literature review could improve the potential role of those ECVs in detecting in vitro resistance in the clinical setting. Unfortunately, and as expected, most publications reported either Etest or SYO data. The validation of these methods as predictors of in vitro resistance could ensure that susceptibility results align with those obtained by the reference methods. However, as for any susceptibility assay, in some instances the commercial ECV test could provide an incorrect classification or an overlapping of results for mutant and WT isolates. For example, the MIC for the mutant is either the same or lower than the ECV instead of being above the ECV result for the particular species and agent. The clinical response to the antifungal agent was introduced whenever such data were reported. Regardless of the promising results summarized below for some species/agent combinations, the ECV does not predict clinical response to therapy, which is the role of the breakpoint.

## 4. Triazoles, Reference High MICs versus Genetic Mutational Changes

Acquired resistance mechanisms can usually be detected using molecular techniques and they can validate either reference or commercial methods ECVs [3,4,29]. Although the most common triazole mechanisms of acquired resistance are the target enzyme gene mutations, these alterations may not always alter the phenotype. This could be because the mutations are ‘silent’ (e.g., do not affect the target site of the enzyme) or the resistance gene is not expressed. However, various molecular mechanisms have been associated with high triazole MICs for *Candida* spp. and poor response to therapy as follows [30]: (i) mutations in the *ERG* genes in combination with modifications in the quantity or quality of the target enzyme (Erg11p); (ii) reduced access of the drug to Erg11p via either MDR (multidrug resistance) or CDR efflux mechanisms; and (iii) an active efflux of azole from the cell through the activation of multidrug efflux transporters encoded by the *MDR* and *CDR* genes [30]. Some of these reports or relationships with reference MIC data are briefly discussed below.

Fluconazole resistant *C. albicans* (CLSI MICs > 64 µg/mL) isolated from human immunodeficiency virus (HIV)-infected patients with oropharyngeal candidiasis were evaluated for resistance mechanisms. Of these isolates, 85% had an overexpression of the *ERG11* gene, as well those encoding the efflux pumps, *MDR1* and *CDR* [31]. *ERG11* gene mutations (Y132F, K143R, F145L, S405F, D446E, G448E, F449V, G450E, and G464S) were also associated with a >4-fold CLSI fluconazole and voriconazole MIC increase among 63 resistant *C. albicans* clinical strains [32]. In 2005, the mechanisms of azole resistance were evaluated in 29 clinical *C. glabrata* strains with CLSI fluconazole MICs ≥16 g/mL (three-year survey); upregulation of *CDR1* and *CDR2* (alone or together) genes were responsible for the resistance to fluconazole [33]. Correlation was documented between high EUCAST fluconazole and voriconazole MICs (>64 µg/mL and >8 µg/mL, respectively) and in vivo data (*Galleria mellonella* model) for two post-treatment *C. tropicalis* clinical isolates; both amino acid substitutions in the *Erg 11* (*G464D* and *Y132F*) and *ERG3* (S113G) genes were reported [34]. For *C. parapsilosis*, fluconazole exposure followed by *ERG11* sequencing led to a *Y132*F substitution as well as *ERG11 CDR1* and/or *MDR1* overexpression [35]. This literature review revealed that azole MIC data by the commercial methods for *Candida* spp. were mostly for isolates harboring mutations in the *ERG 11*, *ERG 3*, or *MRR1* genes (discussed below and listed in Table 3 and Table 4). 

## 5. Echinocandins, High MICs, and Genetic Changes

The mutations responsible for in vitro echinocandin resistance are mostly *FKS1* gene alterations among the most prevalent *Candida* spp.; however, both *FKS1* point mutations and *FKS2* are the most common resistance mechanisms among *C. glabrata* isolates [30]. In the clinical setting, various *FKS1* and/or *FKS2 Candida* mutants with high CLSI echinocandin MICs were reported between 2005 and 2013 [36,37,38,39,40]. During caspofungin clinical trials, 4/5 *C. albicans* and one C. *krusei* with high caspofungin MICs also had various amino acid changes (*Fks1p-S645F* and *S645P*, *R1361G*, respectively) [36]. *C. tropicalis*, causing breakthrough fungemia in 3/37 stem cell transplant patients receiving caspofungin as either prophylactic or primary treatment were reported; these isolates had specific amino acid changes within the *Fks1p* hot spot as well as high echinocandin MICs [37]. Regarding *C. glabrata* infections, it was concluded in three studies that the presence of *FKS* mutations, instead of the MIC, was the better predictor of the clinical response to echinocandin therapy as follows: patients infected with mutants with high echinocandin MICs either did not respond to therapy or experienced an infection relapse [38,39,40]. In addition, caspofungin MICs were not as good predictors of clinical response as anidulafungin and micafungin breakpoints [41].

## 6. Development of Etest and SYO ECVs for *Candida* spp. and Resistance Mechanisms

### 6.1. Etest ECVs: Echinocandins

Between 2015 and 2021, four multi-laboratory studies defined ECVs for both triazoles and echinocandins versus *Candida* and *Aspergillus* spp. [7,8,9,10]. Although only some reports provide data for NWT isolates by the commercial methods, Table 3 and Table 4 list sufficient echinocandin and triazole data for the most prevalent *Candida* spp. During the definition of Etest ECVs for echinocandins in 2016 [8], Etest MICs were reported for a total of 102 (micafungin) to 140 (caspofungin) NWT or molecularly defined echinocandin *Candida* mutants (*FKS* gene mutations [all species] or *FKS2* [*C. glabrata*]) by participant laboratories [8]. Applying the Etest ECVs for the three echinocandins in that study [8], the anidulafungin ECVs would have recognized 92% (107/116) of the mutants (Table 3), while both caspofungin and micafungin were lower performers (105/140 [75%] and 86/102 [84%]). The term “overlap” in Table 3 and Table 4 indicates that the MIC for some of the mutants (9 in the anidulafungin set: shaded mutations) was either the same or lower than the listed ECV. These mutants would be classified as WT, not an uncommon event. Some potential explanations have been given above [7,8,9,10].

In addition to the Etest ECV study data, this review includes anidulafungin data from three other studies: *C. glabrata* (42/42: 100% performance) and for some laboratory mutants (*C. albicans* and *C. krusei:* 7/8) [41,42,43,44]. Results were somewhat less promising in 2/3 studies for *C. albicans*, *C. glabrata*, *C. krusei*, and *C. tropicalis* (15/17 and 3/8) [43,45], but the isolate numbers were small. In the more recent study [45], the *FKS2-S663P* (2 isolates) mutation was evaluated which is among the strongest phenotypes. It is interesting that the modes were higher in that study than the global values in the Etest definition study [8,45].

Etest caspofungin MICs for 38 mutants and 1 WT strain are also summarized in Table 3 [43,46,47]; 85% of these strains would have been recognized by the caspofungin Etest ECVs. Reports were also found for micafungin and the three echinocandins Etest data for 5 and 1 mutants, respectively [48,49]. Echinocandin MICs for these six mutants were above the ECVs [48,49]. In general, anidulafungin Etest MICs appear to recognize most of the *FKS* gene mutants. Further studies should be done to confirm these conclusions.

### 6.2. Etest ECVs: Triazoles

The development of Etest triazole ECVs for *Candida* spp. was more complicated. It took three collaborative studies to obtain some consensus regarding the modal compatibility that has been observed among Etest MICs [9,10,50]. The modal analysis indicated that the most reliable data were the Etest fluconazole MICs. The Etest fluconazole MICs for 66 available mutants are presented in Table 3; a total of 97% mutants could have been recognized by these Etest ECVs, while the percentages were much lower for both voriconazole and posaconazole ECVs [10]. Similar results were reported for 8 fluconazole TAC and CDR laboratory mutants [51]. Again, further studies should support these conclusions.

**Table 3:** Relationship between echinocandin and triazole gene mutations and Etest MICs for NWT isolates (non-wild type: mutants) ^1^.

Species	No. NWT (%)	Agent MICs (µg/mL)	Mutations ^2^	ECV (µg/mL)	Refs.
		**AND**			
*C. albicans*	48/55	≥0.01	^2^*FKS1*-*L644L/stop*, *F641L*, *S645F* (*2 isolates*), *R6471*, *S645P* (2 isolates)	0.01	[8]
*C. glabrata*	36/37	≥0.03	^2^ *FKS2-K1323E*	0.03	
*C. krusei*	14/15	≥0.06	^2^ *FKS1-T6571, L660I*	0.06	
*C. tropicalis*	9/9	≥0.03	*FKS1-F655S*, *F655X*, *F76S*, *S80P*, *V213I*, *V265I*, *S645P/S* (2x), S645S/P	0.03	
Total	107/116 (92%)		^2^ **Overlap**: mostly among 7 of the *C. albicans* mutants		
		**AND**		**ECV**	
*C. glabrata*	10/10	≥0.03	*FKS1-D632H*, *D632Y*, *R635I; FKS2-F659L*(*3x*), *F659 del* (*2x*), *S663P*, *F659S*	0.01	[8,41]
	22/22	≥0.03	*FKS2-F659S* (14x), *F659S/S663A* (7x), *F659S/S663A/D666E*		[8,42]
	10/10	≥0.25	*FKS1p-F625S*, *S629P*, *D632G* *FKS2-F659V*, *F659S*, *S663P*, *S663F*, *D666G*, *D666E*, *P667T*		[8,43]
Total	42/42		**No overlap**		
		**AND**		**ECV**	
*C. albicans*	7/7	≥0.01	Laboratory mutants	0.01	[8,44]
*C. krusei*	0/1	0.03	^2^**Overlap**: *C. krusei*	0.06	
Total	7/8				
		**AND**		**ECV**	
*C. albicans*	9/10	≥0.01	*FKS1-F641S* (2x), *S645Y*, *S645F*, *S645P (3x)*, *S645F*, and *R1361R/H*, *P649H*, *D648Y*	0.01	[8,43]
*C. krusei*	2/3	≥0.06	*FKS1-R1361G*, *F655F/C*, *L658W* and *L701M*, *D700M*, *L701M*	0.06	
*C. tropicalis*	4/4	≥0.5	*FKS1-F76S*, *S80P*, *V213I*, and *V265I*	0.03	
Total	15/17 (88%)		^2^**Overlap**: *C. albicans* and *C. krusei* (1 isolate each)		
		**AND**		**ECV**	
*C. albicans*	1/3	≥0.01	*FKS1*-S645S, 1360R/K (2x)	0.01	[8,45]
*C. glabrata*	2/5	≥0.03	*FKS2*-*L660F*, *S663P*(2x), *L1381S*, *R1377T*	0.03	
Total	3/8		^2^ **Overlap**: both species		
		**CAS**		**ECV**	
*C. albicans*	9/11	≥0.5	*FKS1-F641Y*, *F641S(2x)*, *S645Y*, *S645F*, *S645P (3x)*, *S645F* and *R1361R/H*, *P649H*, *D648Y*	0.5	[8,43,46]
*C. glabrata*	12/15	≥1	*FKS1p-F625S*, *S629P*, *D632G*, *S645P* *FKS2-L644W*, *S645P (2x)*, *F659V*, *F659S* (2x), *S663P*, *S663F*, *D666G*, *D666E*, *P667T*	1	
*C. tropicalis*	5/6	≥1	*FKS1-F641S*(2x), *F76S*, *S80P*, *V213I*, *V265I*	1	
Total	26/32 (81%)		^2^**Overlap**: each species		
		**CAS**		**ECV**	
*C. krusei*	1/1	0.12	WT	0.5	[8,47]
	6/6	1–4	*FKS1-F655L*, *P663Q(3x)*, *F655L*, *S659P/S*		
Total	7/7		**No overlap**		
		**MCA**		**ECV**	
*C. albicans*	1	32	*FKS1-641S*	0.03	[8,48]
*C. glabrata* *C. tropicalis*	1 3	0.12 ≥0.25	*FKS2-AF649* *FKS1-F641L*, *R647G*, *S645F*	0.03 0.12	
Total	5/5		**No overlap**		
		**AND/CAS/MFC**		**ECVs**	
*C. tropicalis*	1	≥1/≥1/0.5	*FKS1-S80P*	0.031/1/0.12	[8,49]
		**FLU**		**ECV**	
*C. albicans*	6/6	≥1	*ERG11: A114S*, *G464S*, *T22OL*, *E66D*, *G448R*, *Y132H*, *T220L*, *V437I*, *F145T*, *V437I*, and *CDR2*/*MDR* overexpression	1	[10,50]
*C. glabrata*	5/5	≥64	*ERG11*: *A114S*, *G464S*	128	
*C. krusei*	2/2	≥16	*ERG11*: *Y87X* (2x)	16	
*C. parapsilosis*	45/45	≥2	*ERG11*: *Y132F*, *A114S*, *G464S*	2	
*C. tropicalis*	6/6	≥4	*ERG11*: *K143R*, *Y132F*, *G464D*	4	
*C. guilliermondii*	0/2	≥16	*ERG11*: *A114S*, *G464S*	16	
Total	64/66 (97%)		^2^**Overlap**: *C. guilliermondii*		
		**FLU**		**ECV**	
*C. albicans*	1	1	WT	1	[10,51]
Total	8/8	8–16	*TAC* and *CDR1* lab. Mutants **No overlap**		

^1^ The Etest fluconazole *C. glabrata* ECV was tentative at 64 µg/mL [10]; NWT, non-wild type, or mutants: MICs above the MIC. ^2^ Mutations present in isolates for which the Etest MIC was available; overlap: mutant isolates classified as WT (shaded mutations) when the MIC is below the ECV for that combination of method, agent, and species.

**Table 4:** Relationship between triazole and echinocandin gene mutations and SYO MICs for NWT isolates (mutants) ^1^.

Species	No. NWT (%)	Agent MICs (µg/mL)	^2^ Mutations	ECV (µg/mL)	Refs.
		**POS**	***ERG11*** **mutations**		
*C. albicans*	59	≥0.06	^2^ *V112I/G450R, D116E/K128T/V159I* (3x)	0.06	[9]
**Total**	55/59 **POS (93%)** **FLU** (81%)		^2^ **Overlap**		
		**FLU**	***ERG11*** **mutations**	**ECV**	
*C. parapsilosis*	49	>2	*T591C* (2x), *R398I* (2x), *Y132F/T591C* (24x),	2	[9,52]
			*Y132F/T591C* (19x),		
			*Y132F/T591C/G398I* (2x)		
**Total**	49/49		**No overlap**		
		**FLU**	***MRR1* mutations**	**ECV**	
*C. parapsilosis*	29	≥2		2	[9,52]
Total	26/29		*G53A/G1214A (R405K), 1 nt del at 1331*, G53A/C744T (2x), *G1747A (G583R)* (3x),		
			*C1856T (A619V)* (6x), *G53A* (9x), *G1436A* *(G583R)* (5x), *G53A/C1856T/G1214A* *(R405K)* ^2^ **Overlap**		
**Total**	**75/78** **(96%)**				
		**FLU**	***ERG 11***	**ECV**	
*C. tropicalis*	1/5	0.5–>128	*T225C*, *G264A*, *G1362A*, *T1554C* **^2^** **Overlap**	4	[9,53]
			***ERG3***		
	**3/3**	>128	2-bp insertion in positions 1130 and 1131 **No overlap**		
**Total**	**4/8**				
		**MCA**		**ECV**	
*C. albicans*	38/41	≥0.06	^1^ *FKS1 L644L/stop, F641L, R136IR/H, S645F* (2x).	0.06	[7]
*C. glabrata*	24/26	≥0.03	^1^ *FKS2 F659L *(3x)*, F659S, S663P*	0.03	
*C. krusei*,	5/5	>0.25	^1^ *FKS1 H675H/Q*	0.25	
*C. tropicalis*	8/8	>0.06	^1^ *FKS1 F641L, F641S*	0.06	
**Total**	76/81 **(94%)**		One *C. dubliniensis*: not listed ^2^ **Overlap**		
		**CAS**		**ECV**	
*C. glabrata*	5	0.12–8	*FKS1-1634V* (2/3 responses); *F625C* (1/1 response), *S629P* (persistent)	0.25	[7,54]
	5	0.5–8	*FKS2-S663P* (4/4 persistent), *S663F* (1/1 response)		
**Total**	10		^2^ **Overlap: first set of mutants**		
*C. auris*	11	**FLU**: Resistant Cutoff: ≥32	***ERG 11***: *Y132F*, *VF125AL*, *K117R*, *N335S*, *E343D*,	NA	[55]
			***MR11***: *N647T*, *TAC1b-S195G*, *A651P*, *A657V*,		
			*FKS1HP1-S639P*,		
			***ERG3***: *S58T*		

^1^ Mutations present in isolates for which the SYO MIC was available. ^2^ Overlap: mutant isolates classified as WT (shaded mutations) or when the MIC is below the ECV for that combination of method, agent, and species [7]. NA: not available.

### 6.3. SYO ECVs: Triazoles

Regarding the performance of SYO ECVs for the triazoles, the SYO posaconazole ECV of 0.06 µg/mL recognized the highest percentage of *C. albicans* mutants (55/59; 93%) (Table 4), followed by the itraconazole ECV of 0.12 µg/mL (53/59; 90%), the voriconazole ECV of 0.01 µg/mL (52/59; 88%), and the fluconazole SYO ECV of 1 µg/mL (48/59; 81%) [9]. These *C. albicans* mutants had the following *ERG11* substitutions: F145L, Y132H, S442F, S405F, G464S, A114S, G464S, F145T, T22OL, and P98A (alone or in combination). Although high CLSI triazole MICs have been documented for most of those substitutions [6,8,33,34,35], T22OL and P98A (alone or in different combinations with E266D, G448R, V437I, V488I, K143R, and Y132H/X) have not been previously reported [9]. The fluconazole data for 78 *C. parapsilosis* isolates, from one of the participant laboratories, are also displayed in Table 4 [9,52]. The SYO fluconazole MICs for the 49 *C. parapsilosis ERG11* mutants and for 26/29 of the *MRR1* mutations, respectively (96%), was >2 µg/mL (above the ECV for this species). 

A report of fluconazole SYO data for *C. tropicalis* isolates having *ERG 11* and *ERG 3* mutations is also listed [53] (Table 4). These strains were associated with high azole resistance following prolonged therapy for urinary candidiasis in a dog. Pre- and post-azole treatment isolates had identical silent mutations in the *ERG11* gene, but the latter displayed increased triazole MICs. For example, the fluconazole SYO ECV for this species was 4 g/mL and the MICs increased from ≤4 µg/mL to >128 µg/mL or switched from WT to non-WT, as also detected by the CLSI method [53].

### 6.4. SYO ECVs: Echinocandins 

In the report of the development of echinocandin ECVs for the SYO method for *Candida* spp. Micafungin SYO MICs data for 81 molecularly defined *Candida* spp. Mutants (*FKS1* and *FKS2* gene mutations) were evaluated [7]. The number of NWT varied from 41 *C. albicans* to 26 *C. glabrata*, 8 *C. tropicalis*, and 5 *C. krusei* (Table 4). When applying the method specific SYO ECVs, the following percentages of NWT would have been identified: 94%, 89%, and 91% by micafungin, anidulafungin, and caspofungin SYO ECVs, respectively [7]. Only the micafungin data are listed in Table 4. MICs for the WT isolates were within the expected range, but their number was small (only 14). Therefore, in contrast to the Etest ECVs for these agents, the best performer was micafungin, but isolate numbers were low for two of the species.

Another interesting and different report has been listed in this table where 72 patients with *C. glabrata* candidemia were evaluated [54]. Six of them developed candidemia while being treated with an echinocandin and another six with fluconazole. A total of 10/72 isolates had *FKS* mutations as listed in Table 4 (clinical response with caspofungin treatment in 4 of 10 patients and relapse or nonresponse in the other 6 patients). The SYO ECV for *C. glabrata* is 0.25 μg/mL, so some overlap was observed [7]. These are promising results, as the percentage of *C. glabrata* mutants that were recognized by the SYO ECV for this species was 92%, or 24/26 of the mutants evaluated (Table 4) [7,54].

#### *Candida auris*: Data for Mutants

Although no ECV is available for *C. auris* versus any antifungal agent, SYO data for mutants were reported from South Africa for this species [55]. Eighty-four *C. auris* isolates (91%) were resistant to at least one antifungal agent; both resistant and susceptible isolates had mutations. Common substitutions had SYO fluconazole MIC of ≥32 μg/mL and are listed for 11 mutants in Table 4.

## 7. Conclusions

Only ECVs for the Etest and SYO methods and the most common *Candida* spp. are available versus the echinocandins and triazoles. Although the number of mutants used to evaluate the accuracy of those ECVs detecting mutants (NWT) is sometimes low, some satisfactory conclusions can be made: (i) for the Etest method, the best performers were anidulafungin ECVs (92% for the common species, >98% for *C. glabrata*) and fluconazole ECVs (97%, mostly for *C. parapsilosis*); (ii) by the SYO, posaconazole ECVs recognized 93% (versus 81% by the fluconazole ECV) of the *C. albicans* and 96% of the *C. parapsilosis* NWT isolates, while micafungin ECVs recognized 94%, mostly *C. albicans* and *C. glabrata*. Smaller sets, some with clinical data, were also listed for other combinations in Table 3 and Table 4. These are noteworthy results for the clinical use of both commercial methods to identify antifungal resistance (NWT isolates). However, there is the need to define ECVs for other species, including the *C. neoformans* complex and emerging species. It is important to note that the ECV does not predict clinical response as the BP does; therefore, when the BP is available for a species/agent combination, that categorical endpoint should be used.

## Figures and Tables

**Table 1:** Method-dependent ECVs of three triazoles and three echinocandins for species of *Candida* by three susceptibility testing methods ^1^.

Species ^3^				Agent/Method-Dependent ECVs (µg/mL) ^2^													
	FLU			POS			VOR			AND			CAS		MCA		
	SYO	Etest	CLSI	SYO	Etest	CLSI	SYO	Etest	CLSI	SYO	Etest	CLSI	SYO	Etest	SYO	Etest	CLSI
*C. albicans*	1	1	0.5	0.06	0.06	0.06	0.01	0.03	0.03	0.12	0.01	0.12	0.25	0.5	0.06	0.03	0.03
*C. dubliniensis*	1	0.5	0.5	0.12	NA	0.12	0.01	1	0.03	0.25	NA	0.12	0.25	NA	0.12	NA	0.12
*C. glabrata*	64	64	8	4	NA	1	2	1	0.25	0.12	0.03	0.25	0.25	1	0.03	0.03	0.03
*M. guilliemondii*	16	16	8	1	NA	0.5	0.5	0.5	0.12	4	NA	8	2	NA	2	NA	2
*P. kudriavzevii*	128	128	32	1	NA	0.5	1	1	0.5	0.25	0.06	0.25	1	1	0.25	0.25	0.25
*C. kefyr*	NA	1	1	NA	NA	0.5	NA	NA	NA	NA	NA	0.25	NA	NA	NA	NA	0.12
*C. lusitaniae*	4	2	1	0.12	NA	0.06	0.03	0.03	NA	0.25	NA	1	1	NA	0.12	NA	0.5
*C. parapsilosis SC*	2	2	1	0.12	0.12	0.25	0.01	0.25	NA	4	8	4	2	4	4	2	2
*C. parapsilosis SS*	2	NA	NA	0.25	NA	NA	0.03	NA	NA	NA	NA	NA	NA	NA	NA	NA	NA
*C. tropicalis*	4	4	1	1	0.25	0.12	0.5	0.25	0.12	0.5	0.03	0.12	0.25	1	0.06	0.12	0.06

^1^ Although some ECVs from the different methods are the same for certain species, in some instances they are >2 dilutions different. FLU: Fluconazole; POS: Posaconazole; VOR: Voriconazole; AND: Anidilufungin; CAS: Caspofungin; MCA: Micafungin; ^2^ SYO/Etest ECVs based on MICs determined by each commercial method, respectively, and CLSI by broth microdilution methods; the EUCAST ECVs can be found elsewhere, although caspofungin ECVs are only available for some species by the CLSI, but not for the EUCAST [2,4,7,8,9,10]; ^3^ *Candida guilliermondii* (*Meyerozyma guilliermondii*), *C. krusei* (*Pichia kudriavzevii*), *C. kefyr* (*Kluyveromyces marxianus*), *C. lusitaniae* (*Clavispora lusitaniae*), *Candida parapsilosis* species complex and sensu stricto; NA: not available or applicable.

**Table 2:** Some of the available commercial methods ^1–7^ for antifungal susceptibility testing: in certain studies, CA was evaluated prior to the development of current reference BPs ^8^.

Method	Brief Description
^1^ SYO ^®^; TREK	Sensititre colorimetric plate Microdilution plate wells are dosed with the antifungal appropriate dilutions, as well as with the colorimetric indicator. MIC: lowest antifungal concentration showing no color change (no growth) following 24–48 h of incubation.
^2^ Etest ^®^	Gradient concentration methods ^®^
^3^ Biomerieux Liofilchem ^®^	Based upon a continuous concentration gradient of drug infused on a plastic non-porous strip. The agent diffuses into an agar plate and the MICs are where the growth intersects with the testing strips after 24 to 48 h or until the susceptibility ellipse is created.
^4^ Automated Vitek-2 ^®^	Automated system that spectrophotometrically and simultaneously provides the isolation, identification, and the MIC results of the pathogen.
^5^ Neo-Sensitabs tablets ^®^ and disk diffusion	The 9-mm tablets contain the following concentrations: amphotericin B (10 μg), caspofungin (5 μg), fluconazole (25 μg), itraconazole (8 μg), and voriconazole (1 μg). Discs can also be used when available (see CLSI M44 method).
^6^ ATB ^®^ fungus panel	This microdilution non-colorimetric panel determine MICs to six antifungal agents.
^7^ The Fungitest ^®^	A microdilution colorimetric breakpoint method for amphotericin B, flucytosine, fluconazole, itraconazole, ketoconazole, and miconazole. Not much since BPs are not available for most of these antifungals.

^1^ Sensititre YeastOne panel/plate, TREK Diagnostic Systems, USA; ^2^ Etest, bioMerieux Marcy-l’Etoile, France; ^3^ Liofilchem, Roseto degli Abruzzi, Italy; ^4^ Automated Vitek-2, Biomerieux, France; ^5^ Neo-sensitabs tablets, A/S Rosco, Taastrup, Denmark; ^6^ ATB ^®^ Fungus panel, Biomerieux, France; ^7^ Fungitest ^®^, Sanofi Diagnostics Pasteur, Paris, France. ^8^ Early reports of both EA and CA agreement between reference BPs and commercial methods from 2003 to 2011 [19,20,21,22,23,24,25,26].

## Data Availability

Only published data were reported as noted.
